# A persistent sink reservoir as a potential source of *Pseudomonas aeruginosa* infections in pediatric oncology patients

**DOI:** 10.1017/ash.2025.54

**Published:** 2025-03-21

**Authors:** LaTasha Richards, Craig Gilliam, Jessica Brazelton, Heather L. Glasgow, Randall T. Hayden, Hana Hakim

**Affiliations:** 1 Department of Infection Prevention and Control, St. Jude Children’s Research Hospital, Memphis, TN, USA; 2 Department of Pathology, St. Jude Children’s Research Hospital, Memphis, TN, USA; 3 Department of Infectious Diseases, St. Jude Children’s Research Hospital, Memphis, TN, USA

## Abstract

**Objective:**

Outbreaks of *Pseudomonas aeruginosa* infections have been linked to water-related sources. We describe the investigation of a suspected outbreak of five *P. aeruginosa* infections in pediatric oncology patients in 2021 that triggered a retrospective review and prospective monitoring of additional cases, environmental sampling, and bacterial genomic analysis.

**Setting and patients:**

Pediatric oncology center.

**Methods:**

Medical records of patients with *P. aeruginosa* were reviewed and staff were interviewed to identify common exposures. Environmental samples were cultured for *P. aeruginosa.* Patient and environmental isolates underwent whole genome sequencing and core genome multi-locus sequence typing (cgMLST) and sequences were added to a previously existing library of *P. aeruginosa* clinical isolates collected in 2017 and onwards to determine strain relatedness.

**Findings:**

During 2019–2022, 82 patients with 110 episodes of *P. aeruginosa* infections were identified and 132 isolates of *P. aeruginosa* were sequenced. Twenty-three environmental samples were collected, of which two grew *P. aeruginosa* in culture. CgMLST demonstrated four multi-patient isolate clusters but no genetic relatedness among the isolates from the patients in the suspected outbreak. Two sink-derived isolates from 2021 were genetically related to patient-derived isolates from 2018 and 2017.

**Conclusions:**

Sequencing revealed there is no common source or linkage between the isolates of the suspected *P. aeruginosa* outbreak in 2021. However, it revealed genetic relatedness of previous patient strains to later strains collected from hospital sinks, suggesting persistent colonization of a reservoir with *P. aeruginosa.*

## Introduction

*Pseudomonas aeruginosa* is a common cause of healthcare-acquired infections (HAI) and is associated with significant morbidity and mortality, especially in immunocompromised patients. Several outbreaks of *P. aeruginosa* infections have been linked to a water-related source such as bath toys,^1^ contaminated artificial tears,^2,3^ whirlpool bathtub,^4^ sink traps,^5,6^ surface cleaning solution,^7^ among others. The fact that moist environments frequently serve as a reservoir of *P. aeruginosa* is related to this organism’s ability to survive and persist for a long time in the biofilms of water systems rendering it difficult to eradicate by disinfectants.^8^

In February 2021, an increase in the number of patients admitted with *P. aeruginosa* infections was identified by the Infection Prevention and Control Department (IPC) at St. Jude Children’s Research Hospital (SJCRH). It was prudent to investigate these infections as a suspected outbreak because patients frequently return to receive care either in inpatient units or outpatient care units at SJCRH such as specialty clinics, acute care clinic, or infusion center. The objective of this report is to describe the findings of the investigation of the suspected outbreak of *P. aeruginosa* infections in pediatric oncology patients, the environmental source testing, and whole genome sequencing of *P. aeruginosa* isolates for genetic relatedness.

## Methods

### Study design and patients

In February 2021, an increase in the positive clinical cultures reporting *P. aeruginosa* isolates from specimens collected in the outpatient units at SJCRH over a 2-month period was recognized, which triggered an investigation of a suspected outbreak. SJCRH is a 78-inpatient-bed specialized referral center for children with cancer and other catastrophic diseases. Cases were defined as patients with *P. aeruginosa* isolated in clinical specimens collected from hospitalized patients in inpatient units or patients seen in outpatient units at SJCRH starting on January 1^st^, 2021. Medical records of patients were retrospectively reviewed for clinical data such as demographics, clinical course, hospitalization, treatment, outcomes, exposures, procedures, medications, and location of healthcare to identify common factors and a potential source. Clinical staff who provided care to the patients and supportive staff such as environmental services and facility management staff were interviewed. For a historic baseline and additional case finding, a report of all clinical cultures that isolated *P. aeruginosa* from January 1^st^, 2019 to February 28^th^, 2021 was generated. For prospective monitoring, this report was regularly updated to identify new cases until December 31^st^, 2022. HAI are nosocomially acquired infections that are not present or incubating at the time of hospitalization. Operationally, HAI was defined as an infection with onset on or after the third calendar day of hospitalization until one-day post-discharge. Infections that did not meet these criteria were classified as community-acquired infection (CAI). *P. aeruginosa* that was isolated from non-sterile body sites only such as stool, peri-anal surveillance swabs, sputum, or tracheal aspirate specimens without any other associated cultures positive for *P. aeruginosa* was considered to represent colonization. For patients with repeated positive cultures, a new episode was determined if the clinical signs and symptoms of the previous episode resolved, subsequent cultures were negative (if applicable) on appropriate antibiotic treatment, and at least 2 weeks have passed since the onset of the previous episode of infection. This study was approved by the institutional review board at SJCRH.

### Clinical and environmental samples

Based on the investigation findings, targeted environmental specimens were collected and selectively cultured for *P. aeruginosa* at a certified environmental laboratory (Aerobiology Laboratory Associates, Inc, Virginia, USA). A volume of 250 ml of water was collected for cultures using a sterile container. Sponge swab specimens were collected as described in the Supplementary Table S1. To increase the yield, the swab was placed into 1 ml sterile water and was vortexed. Next, 0.1 ml, 0.01 ml, and 0.001 ml of the samples were plated on blood agar plates for culture. *P. aeruginosa* isolates were identified to the species level and sub-culture plates were sent to the Microbiology and Molecular Microbiology laboratories at SJCRH for whole genome sequencing. Clinical specimens collected from patients receiving routine clinical care in the inpatient units or outpatient units are processed for cultures at the same laboratory at SJCRH. *P. aeruginosa* isolates from the clinical specimens from January 2017 to February 2021 had been included in a routine genomic surveillance program at our hospital as previously described^9^ and their sequences had been previously defined as part of that program. *P. aeruginosa* isolates from March 2021 to December 2022 were prospectively sequenced irrespective of epidemiologic links, part of the genomic surveillance program along with environmental isolates and previous clinical isolates.

### Sequencing of Pseudomonas aeruginosa isolates

Relatedness among all clinical and environmental isolates was evaluated using a core genome multi-locus sequence typing (cgMLST) pipeline from Ridom SeqSphere+ (Ridom Bioinformatics^©^, Germany), which included 4073 core targets and 1444 accessory targets. Strain PAO1 (NC_002516.2) was used as the reference genome and 32 query genomes were included in the task template. Isolates with at least 95% of the defined cgMLST targets were analyzed for relatedness and those with ≤12 different alleles to the nearest neighbor in the cgMLST target gene set were considered highly related (and termed a cluster).^10^ A minimum spanning tree was created using 5517 columns for distance calculation, pairwise ignoring missing values, and plotted on a logarithmic scale.

### Statistical analysis

Patient characteristics were summarized using percentages for categorical data and medians and ranges for continuous data.

## Results

### Description of the suspected outbreak of patients with Pseudomonas aeruginosa infections

Five patients with *P. aeruginosa* infections in January and February 2021 were identified in a suspected outbreak (Table 1). Four of the 5 patients were younger than six, had brain tumors, and were receiving chemotherapy. Two of these four patients had perineal lesions consistent with ecthyma gangrenosum. The fifth patient was 18 years old, day 127 post allogeneic hematopoietic cell transplant for acute myeloblastic leukemia, and had a history of 2 previous infections with *P. aeruginosa*. There were no deaths related to these infections (Table 1). Upon review of medical records for common exposures, previous hospitalization in inpatient unit A was identified as a common factor among four of the five patients. For additional case finding, patients identified with *P. aeruginosa* in December 2020 were also reviewed (Table 1).

**Table 1.** Characteristics of the five patients included in the suspected outbreak of *Pseudomonas aeruginosa* infections and of the five patients reviewed for additional case finding

	Age in years/Sex	Primary Diagnosis	Presentation	Antibiotic Susceptibility	Infection Site	Classification of Infection or Colonization	Hospitalized due to Event	Central Venous Catheter	Neutropenia	History of Previous *P. aeruginosa* Infections or Colonization	Previous Hospitalization in the Inpatient Unit A; Days since Last Time in Unit A	Outcome
Patients of Suspected Outbreak in January and February												
Patient 1	1.0/F	High grade glioma of brain stem	FN	Pan-susceptible	Bacteremia; Perineal cellulitis	CAI	Yes	Yes	Yes	No	Yes; 9 days	Recovered
Patient 2	1.2/M	Medulloblastoma	FN	Pan-susceptible	Bacteremia	CAI	Yes	Yes	Yes	No	Yes; 7 days	Recovered
Patient 3	3.9/F	Embryonal brain tumor	FN	Pan-susceptible	Bacteremia	CAI	Yes	Yes	Yes	No	Yes; 9 days	Recovered
Patient 4	5.7/F	Medulloblastoma	FN	Intermediate susceptibility to cefepime; Otherwise, susceptible	Bacteremia; Perineal cellulitis	CAI	Yes	Yes	Yes	No	Yes; 24 days	Recovered
Patient 5	18.1/M	Acute myeloblastic leukemia	FN	Pan-susceptible	Bacteremia	CAI	Yes	Yes	Yes	Yes, sinusitis in August 2020, and bacteremia in October 2020	No	Recovered
Patients in December Reviewed for Additional Case Finding												
Patient 6	7.2/M	Neuroblastoma	FN	Pan-susceptible	Cellulitis	HAI	NA	Yes	Yes	No	Yes; 105 days	Recovered
Patient 7	4.1/M	Astrocytoma	Fever	Intermediate susceptibility to ceftazidime and levofloxacin; Otherwise, susceptible	NA	Tracheal Colonization	Yes	Yes	No	No	Yes; 25 days	Recovered
Patient 8	5.5/M	Neuroblastoma	FN	Intermediate susceptibility to ceftazidime and levofloxacin; Otherwise, susceptible	Bacteremia	CAI	Yes	Yes	Yes	No	Yes; 9 days	Recovered
Patient 9	9.1/M	Acute lymphoblastic leukemia	Bilateral ear drainage	Pan-susceptible left ear isolate; Right ear isolate resistant to fluoroquinolones; Otherwise, susceptible.	Chronic ear infection	CAI	No	No	No	Yes, ear infection in October 2020	No	Recovered
Patient 10	12.2/F	Acute lymphoblastic leukemia	Fever, urinary symptoms	Intermediate susceptibility to levofloxacin; Otherwise, susceptible	Urinary tract infection	HAI	NA	Yes	No	No	No	Recovered

F, female; M, male; FN, fever and neutropenia; Pan-susceptible, susceptible to aminoglycosides, fluoroquinolones, cefepime, ceftazidime, meropenem, and piperacillin/tazobactam; HAI, hospital-acquired infection; CAI, community-acquired infection.

### Investigation of an environmental source

Investigation to identify a potential waterborne source included review of the water supply and sewage drain maps of inpatient unit A. Unit A has 20 single-patient rooms. Each patient room has its separate patient bathroom and is connected to an adjacent parent room with its separate parent bathroom (Supplementary Figure S1). All sinks had traps on their drains. The cold water was directly supplied from the city via a loop system around the perimeter of the building and dropped down into each room individually. The hot water came from a single source water heater located in the penthouse and designated for this unit. The hot water temperature was maintained at about 120 degrees Fahrenheit. A water management plan is in place to treat, monitor and culture several points of water for *Legionella spp*. In July 2021, targeted environmental specimens were collected for cultures including water from the hot water tank in the hospital penthouse, and sponge swabs from several locations on inpatient unit A; more specifically from the sink faucet and drain inside patient room, the sink faucet and drain and shower head and drain in patient bathroom, the sink faucet and drain and shower head and drain in the parent bathroom, and the sink faucet and drain and ice machine in the unit nutrition room (Supplementary Table S2). All cultures were negative for *P. aeruginosa* except for samples from the nutrition center sink and a parent room sink (Supplementary Table S2).

### Epidemic curve and characteristics of Pseudomonas aeruginosa Infections from 2019 to 2022

During the study period, the IPC team reviewed *P. aeruginosa* infections detected retrospectively between January 2019 and February 2021, and prospectively between March 2021 and December 2022. A total of 82 patients had 110 episodes of infection or colonization with *P. aeruginosa*. Seventeen patients had multiple episodes of *P. aeruginosa* infection or colonization: 9 patients had 2 episodes, 6 patients had 3 episodes, and 2 patients had 4 episodes and 5 episodes, respectively. A total of 132 isolates collected from 105 episodes in 81 patients were available for sequencing. Seventy-six *P. aeruginosa* isolates from 50 patients with 63 episodes were identified from January 2019 to February 2021, and 56 isolates from 34 patients with 47 episodes were identified between March 2021 and December 2022. Two patients had isolates collected in both time periods.

**Figure 1.**
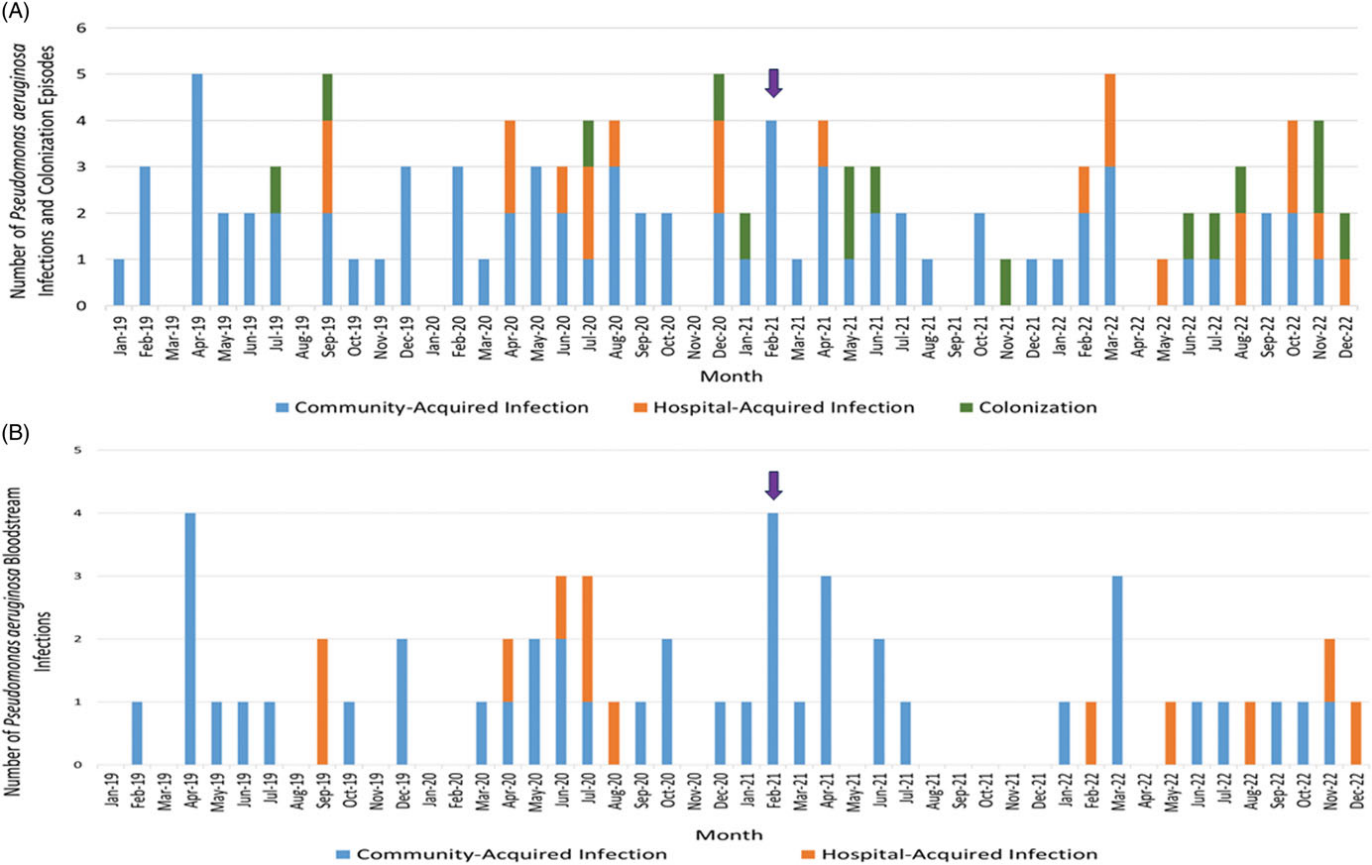
**(A)** Epidemic curve of the number of episodes of *Pseudomonas aeruginosa* infections or colonization from January 2019 to December 2022. **(B)** Epidemic curve of the number of *Pseudomonas aeruginosa* bloodstream infections from January 2019 to December 2022. The purple arrow indicates when the suspected outbreak was identified. Blue bars indicate community-acquired infections, orange bars indicate hospital-acquired infections, and green bars indicate colonization.

Figure 1 shows the epidemiologic curve of the number of *P. aeruginosa* infections and colonization (Figure 1A), as well as bloodstream infections (Figure 1B) from January 2019 to December 2022. Seventy-four (67%) episodes were community-acquired, and 21 (19%) episodes were HAIs. In the remaining 15 (14%) episodes, *P. aeruginosa* was considered to represent colonization. The characteristics of patients with *P. aeruginosa* infections or colonization are summarized in Table 2.

**Table 2.** Clinical characteristics of patients identified to have *Pseudomonas aeruginosa* infections or colonization from 2019 to 2022

Characteristics	Results
Number of patients	82
Number of episodes	110
Median age in years at episode (range)	9.0 (0.3–24.7)
Male sex, N (%)	44 (40)
Primary diagnosis, N (%)	
Leukemia/Lymphoma	36 (33)
Solid Tumor	52 (47)
Brain/Spine Tumor	11 (10)
Hematologic/Metabolic disorders	11 (10)
Transplant recipients, N (%)	24 (22)
Specimen source of isolate^a^, N (%)	119
Blood culture	55 (46)
Urine culture	20 (17)
Abscess/skin culture	21 (18)
Respiratory culture	14 (12)
Stool culture	5 (4)
Ear and sinus drainage culture	4 (3)
Classification of episode, N (%)	
Community-acquired	74 (67)
Healthcare-associated	21 (19)
Colonization only	15 (14)

a Total number of positive cultures is more than the number of episodes because a patient may have positive cultures from more than one specimen source.

### Sequencing of Pseudomonas aeruginosa clinical and environmental isolates

**Figure 2.**
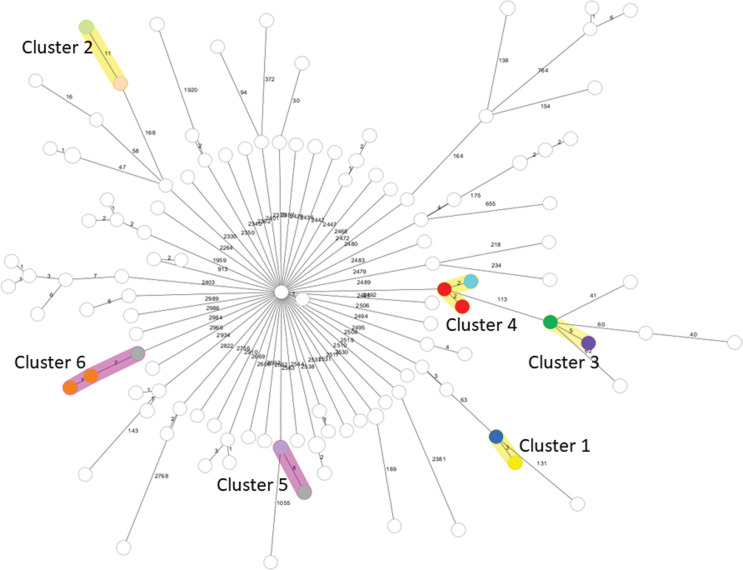
Minimum spanning tree of sequenced *Pseudomonas aeruginosa* isolates in core genome multi-locus sequence type (cgMLST) profiles. Each circle represents a single cgMLST profile. The number of different alleles between cgMLST profiles in a pairwise comparison is shown on connecting lines. Allelic differences below the threshold for a cluster (12) involving multi-patient isolates or patient-environmental isolates are considered genetically related and are labeled as a cluster. Genetically related isolates from the same patient and isolates that are not genetically related are not colored. Isolates involved in a multi-patient cluster are indicated with colors. Cluster 1 involves 2 isolates from 2 patients in 2019. Cluster 2 involves 2 isolates from 2 patients in 2020. Cluster 3 involves 2 isolates from 2 patients in 2020 and 2021. Cluster 4 involves 3 isolates from 2 patients in 2022. Cluster 5 involves 1 patient isolate in 2017 and an environmental isolate in 2021. Cluster 6 involves 3 isolates from one patient in 2018 and an environmental isolate in 2021.

Genetic relatedness of isolates was indicated on the minimum spanning phylogenetic tree as clustering (Figure 2, Table 3). Clustering of isolates from the same patient is expected. Among all sequenced isolates, 4 multi-patient clusters and 2 environmental-patient clusters were identified. The isolates collected from the 5 patients in the suspected outbreak were not genetically related to each other, to any other patient isolates, or to any environmental isolates and thus the outbreak was refuted (Figure 2).

**Table 3.** Characteristics of the four multi-patient clusters of *Pseudomonas aeruginosa* infections and the two clusters involving environmental isolates

Cluster	Number of patients involved	Number of isolates involved	Types of specimens and year of collection	Days between first and last isolate in cluster	Number of allele difference
Multi-patient Clusters					
Cluster 1	2	2	1 blood, 1 stool; both in 2019	38	3
Cluster 2	2	2	Both blood in 2020	160	11
Cluster 3	2	2	Both urine; 1 in 2020, 1 in 2021	501	5
Cluster 4	2	3	1 tissue from one patient, 2 skin from the second patient; all 3 isolates in 2022	0 and 263	2 and 2
Environmental-Patient Clusters					
Cluster 5	1	2	1 blood in 2017; 1 nutrition room sink in 2021	1,489	8
Cluster 6	1	4	1 blood and 2 skin from same patient in 2018; 1 parent room sink in 2021	1,013	0, 1, 7

### Description of genetically related clusters of patients with Pseudomonas aeruginosa infections or colonization

Table 3 provides a summary of the four multi-patient and two environmental-patient clusters of *P. aeruginosa* isolates that were identified as genetically related. Two isolate clusters from multiple patients were identified retrospectively; cluster 1 involved two isolates from two patients in 2019 collected 38 days apart (one isolate was from blood culture from the first patient and one isolate was from stool surveillance culture from the other patient). Cluster 2 involved two isolates from two patients in 2020 (both were blood isolates, collected 160 days apart). Two additional multi-patient clusters were identified prospectively after the suspected outbreak; cluster 3 involved two isolates from two patients in 2020 and 2021, respectively (both were urine isolates collected 501 days apart). Cluster 4 involved 2 patients with isolates from tissue cultures collected in the interventional radiology suite on the same day in 2022, one was from lung biopsy culture and the other was from gastrostomy stoma tissue culture (Table 3). The patient with *P. aeruginosa* isolated from stoma tissue also had an eye drainage swab culture collected 263 days later that also identified a closely related isolate of *P. aeruginosa*. The two environmental isolates from sinks that were collected in 2021 were genetically related to clinical isolates from 2018 and 2017 (Figure 2, Table 3), which possibly indicates a reservoir of *P. aeruginosa* involved in patient infections years earlier (Table 3). In cluster 5, we identified a patient with *P. aeruginosa* isolated in 2017 that was genetically related to an isolate collected from the sink of the nutrition center on inpatient unit A in 2021. The patient had never been hospitalized in that unit. In cluster 6, another patient had *P. aeruginosa* isolated from blood and skin lesion cultures in 2018. Both isolates were genetically related to each other and to an isolate from the sink of the parent room collected in 2021. This patient had been hospitalized multiple times on inpatient unit A. One hospitalization occurred in the patient room connected to the same parent room approximately 2 months prior to developing an infection with *P. aeruginosa*. None of the 10 patients included in these clusters had *P. aeruginosa* isolates from other cultures that were not genetically related.

### Infection prevention and control interventions

Immediately upon suspicion of a potential outbreak and while waiting for the sequencing results, the IPC team started observing cleaning practices of ice machines, patient and parent rooms, nutrition center, and other infection prevention practices on inpatient unit A. The IPC team performed various infection prevention activities including routine rounding on units to observe hand hygiene, cleaning and disinfection of equipment; met with nursing leaders and shared updates regarding infections during staff huddles; provided just-in-time feedback to nursing and environmental services staff if opportunities were identified. There were no breaches identified on inpatient unit A.

## Discussion

In this study, we reported the findings of an investigation of a clinically suspected outbreak of *P. aeruginosa* infections using whole genome sequencing. All clinical isolates involved in the suspected 2021 outbreak were genetically unrelated, thus refuting the outbreak. Genetically related clusters of multi-patient clinical isolates were identified, of which none were epidemiologically suspected. Environmental isolates collected in 2021 were genetically related to clinical isolates collected in 2017 and 2018, which was an unexpected finding that could not be explained in one of the clusters. However, a persistent environmental reservoir is possible.

Environmental sources have been implicated in several previously reported outbreaks of *P. aeruginosa*. Most recently, in February 2023, an outbreak of extensively drug-resistant *P. aeruginosa* eye infections related to the use of contaminated artificial tears was reported in multiple states in the United States.^2,3,11^ Sundermann et al used a genomic surveillance and control program to detect 3 *P. aeruginosa* isolates from two patients that had been previously collected and sequenced and were later found to be genetically related to the isolates from the contaminated artificial tears outbreak.^11^

Among environmental reservoirs, water drainage systems^12–15^ and specifically sink traps have been reported to be a major source of transmission of resistant organisms including carbapenemase-producing *P. aeruginosa.* ^5,16–19^ Regev-Yochay et al used pulsed-field gel electrophoresis and sequencing to characterize bacterial isolates from longitudinally sampled sink drains and outlets in hospital rooms along with patient weekly surveillance isolates of carbapenemase-producing Enterobacterales (CPE).^5^ They found that 24% of patient room sinks were contaminated with CPE and that persistent contamination for more than one year by a dominant CPE strain was common.

We have identified two clusters involving current environmental isolates genetically related to historic clinical isolates collected several years prior. The detection of isogenic isolates from a current environmental source and from historic patient infections suggests a potentially persistent environmental source that had not been tested previously and that could have explained the missing epidemiologic link between the clustering clinical isolates. *P. aeruginosa* can survive in biofilms of a water system for long periods.^8,20^ Previous studies have reported the persistence of environmental strain contamination as reservoirs for more than a year.^4,5,21^ Johnson et al reported the findings of an investigation of an outbreak of clinical multi-drug-resistant *Sphingomonas koreensis* infections proven to be linked to hospital sinks using whole genome sequencing.^21^ The authors identified 12 clinical *S. koreensis* isolates from 2006 to 2016 that were genetically related to environmental sink isolates collected in 2016 and 2017 suggesting the persistence of a clonal strain in a reservoir in hospital plumbing over an eleven-year period.^21^ However, in our investigation, we did not find any clinical isolates that clustered with each of the two environmental isolates from 2021 other than single clinical isolates from 2017 and 2018. If an environmental reservoir persisted over several years, we would have expected to identify additional clinical isolates in each of the two isolate clusters. Another possible speculation about the genetic relatedness of the isolates that were collected several years apart is the paucity of genetic diversity among *P. aeruginosa* strains related to environmental conditions.^22^ Also, the possibility of *P. aeruginosa* biofilm diversity in the hospital water system cannot be excluded and more research about biofilms and diversity among organisms within them is needed.^20,23^

Despite the evidence that implicates sinks as a major source of transmission of outbreak pathogens and the emphasis on the role of sinks in the recently updated practice recommendations for strategies to prevent HAIs through hand hygiene,^24^ decontamination of sinks remains a challenge. Tested interventions such as sink trap and pipe replacement, cleaning with acetic acid, bleach, or hydrogen peroxide, use of foam disinfectants to reduce biofilm formation, and self-disinfecting siphons, among other measures, have frequently failed.^25–32^ Therefore, risk mitigation measures to prevent the spread of the contaminant pathogen to patients have mainly focused on prohibition on use of sinks for disposal of contaminated clinical specimens and fluids, on storing patient supplies away from sinks to prevent contamination from sink water splash-back, and on enhanced hand hygiene practices. Other studies showed that only replacing sinks did not terminate outbreaks and that pathogens can transmit across drainage pipes of the plumbing system, which were not replaced.^19,21,33^ Tsukada et al described an outbreak of multiple CPE bacterial isolates that shared an isogenic plasmid linked to contaminated sinks in a pediatric ward.^19^ The CPE outbreak persisted even after sink replacement and could only be controlled 7 months after implementation of an infection control bundle that focused on sink use and cleaning. Interestingly, eliminating sinks altogether from the hospital patient room environment has also been proposed as a control measure.^16,34^ Removing sinks from patient rooms and implementation of waterless patient care in an intensive care unit terminated a 2.5-year outbreak of carbapenemase-producing *P. aeruginosa* linked to environmental water sources.^16^

There are several limitations to this study. This single-center study evaluated a targeted hospital environment but did not include other locations such as other inpatient units, outpatient care units and housing environments, or other water-based patient care items. Also, evaluation of staff colonization with *P. aeruginosa* was not included. Surveillance testing patients for colonization with *P. aeruginosa* was not performed, which could have missed potential transmission links involving asymptomatic colonized patients. Although genomic sequencing provided evidence for relatedness of clinical and environmental isolates, directionality (patient-to-sink or sink-to-patient) or a network of transmission (eg direct or indirect) cannot be concluded from the findings of this investigation.

In conclusion, this study demonstrated the importance of bacterial genomic analysis for investigating and elucidating unsuspected clusters and evidence of prolonged pathogen colonization on hospital surfaces. This study also emphasizes the significance of proper cleaning and disinfection of sinks to prevent reservoirs of waterborne pathogens such as *P. aeruginosa.*

## Supporting information

Richards et al. supplementary material Richards et al. supplementary material

## Data Availability

Data is available upon request.

## Supplementary material

For supplementary material accompanying this paper visit http://doi.org/10.1017/ash.2025.54. click here to view supplementary material
