# Supplementary material for: A persistent sink reservoir as a potential source of Pseudomonas aeruginosa infections in pediatric oncology patients
Source: Antimicrob Steward Healthc Epidemiol. 2025 Mar 21;5(1):e82. doi: 10.1017/ash.2025.54 (PMC11951235; doi:10.1017/ash.2025.54)
Supplement: Richards et al. supplementary material [file S2732494X25000543sup001.pdf]

Supplementary Materials

Supplementary Table S1. Steps for Environmental Sampling Method

• Two types of samples:<br>○ 250 ml of water from hot water tank collected in sterile container<br>○ Swab cultures of patient room sink, patient bathroom sink, Shower head and drain, parent bathroom sink, parent shower head and drain; nutrition room sink and nutrition room ice machine.
• A 3M ™ Sponge-Stick with 10ml of neutralizing buffer was used to sample for low concertation of *Pseudomonas aeruginosa*.
• Each swab stick was swabbed back and forth in two planes for 10 seconds.
• Swab stick was labelled and placed inside a sealed plastic bag supplied by Aerobiology Laboratory.
• Collection was accomplished by Infection Preventionist performing hand hygiene and wearing exam gloves prior to entering each room for culture collection.

Supplementary Figure S1. The layout plan of a patient room and its connected parent room on Unit A.

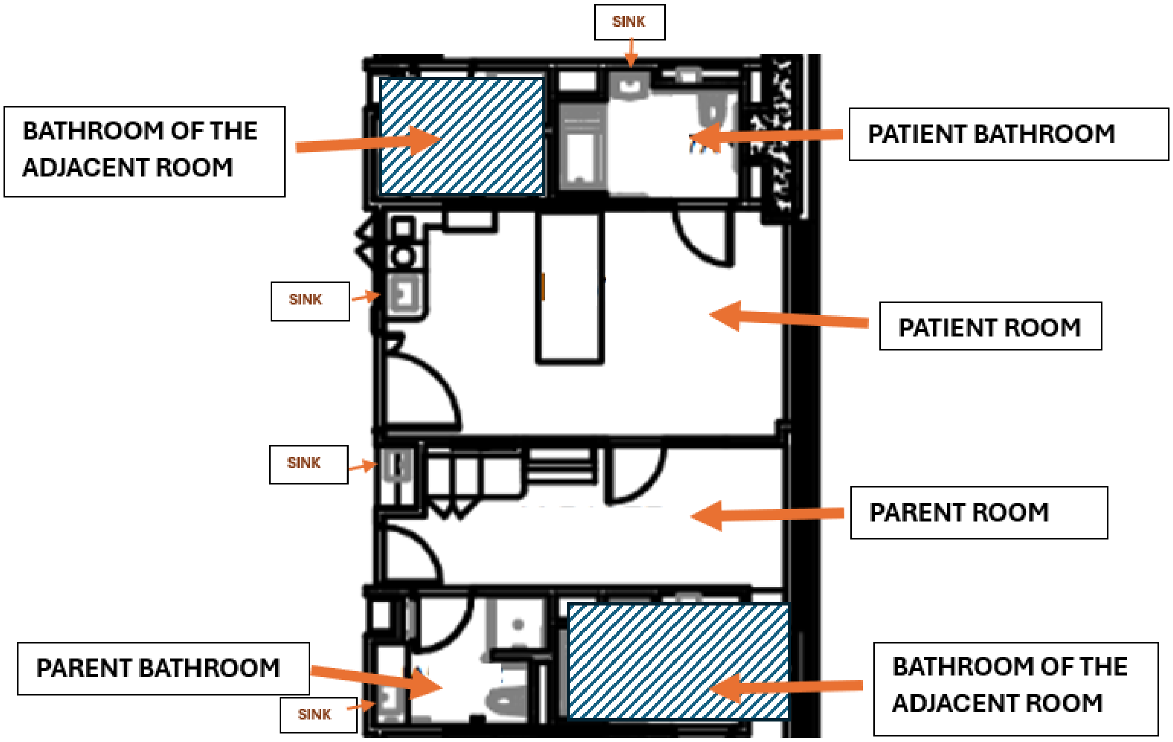

Supplementary Table S2. Location of Environmental Sample Collection and Results of Cultures for *Pseudomonas aeruginosa*

**Location**	**Results**
Potable water in penthouse	No growth
Nutrition room sink	300 CFU/in^2
Nutrition room ice machine	No growth
Sink in patient room 1 (faucet and drain)	No growth
Sink in patient bathroom 1 (faucet and drain)	No growth
Shower in patient bathroom 1 (shower head and drain)	No growth
Sink in parent bathroom 1 (faucet and drain)	30,000 CFU/in^2
Shower in parent bathroom 1 (shower head and drain)	No growth
Sink in patient room 3 (faucet and drain)	No growth
Sink in patient bathroom 3 (faucet and drain)	No growth
Shower in patient bathroom 3 (shower head and drain)	No growth
Sink in parent bathroom 3 (faucet and drain)	No growth
Shower in parent bathroom 3 (shower head and drain)	No growth
Sink in patient room 6 (faucet and drain)	No growth
Sink in patient bathroom 6 (faucet and drain)	No growth
Shower in patient bathroom 6 (shower head and drain)	No growth
Sink in parent bathroom 6 (faucet and drain)	No growth
Shower in parent bathroom 6 (shower head and drain)	No growth
Sink in patient room 7 (faucet and drain)	No growth
Sink in patient bathroom 7 (faucet and drain)	No growth
Shower in patient bathroom 7 (shower head and drain)	No growth
Sink in parent bathroom 7 (faucet and drain)	No growth
Shower in parent bathroom 7 (shower head and drain)	No growth
